# Health benefits of life at moderate altitude: does hypoxia matter?

**DOI:** 10.3389/fphys.2025.1598275

**Published:** 2025-05-13

**Authors:** Johannes Burtscher, Martin Kopp, Max Gassmann, Martin Burtscher

**Affiliations:** ^1^ Department of Sport Science, University of Innsbruck, Innsbruck, Austria; ^2^ Institute of Veterinary Physiology, Vetsuisse Faculty, University of Zurich, Zurich, Switzerland; ^3^ Zurich Center for Integrative Human Physiology (ZIHP), University of Zurich, Zurich, Switzerland; ^4^ Universidad Peruana Cayetano Heredia (UPCH), Lima, Peru; ^5^ Austrian Society for Alpine and High-Altitude Medicine, Innsbruck, Austria

**Keywords:** hypoxia conditioning, moderate altitude, oxygen, health, ageing, life span

## 1 Introduction

Epidemiological studies show that living at moderate altitudes, i.e., 1,000–2,500 m, is associated with beneficial health effects when compared to lower altitudes. Studies, particularly from the Alpine regions (Faeh et al., 2009; Burtscher et al., 2021; Burtscher et al., 2025) and the United States of America (Ezzati et al., 2012; Thielke et al., 2015), unanimously report lower mortality rates, e.g., of all causes, cardiovascular diseases, certain cancers and neurodegenerative diseases in populations living at moderate altitudes. Lifestyle differences such as higher physical activity levels, and environmental factors such as cooler ambient temperatures (particularly in times of accelerated global warming), lower levels of aeroallergens, and elevated solar radiation (possibly via increasing Vitamin D synthesis) might all contribute to healthy ageing when living in those areas (Faeh et al., 2016; Burtscher et al., 2021). In addition, these studies also suggested a protective role of higher altitude-associated hypobaric hypoxia. However, it remains unclear how the small decrease in barometric – and associated partial oxygen – pressure at moderate altitude could provoke beneficial adaptive responses. At least in awake and resting humans, the resulting mild hypoxic conditions are not thought to induce robust hypoxic responses. This opinion article discusses why hypoxic episodes can occur even at moderate altitudes and which effects they may trigger to contribute to the beneficial health outcomes mentioned above.

## 2 Can hypoxia promote beneficial adaptations at moderate altitude?

A major environmental characteristic of increasing altitude is the decreasing atmospheric pressure and the associated hypobaric hypoxia. In principle, exposure to hypoxia can trigger adaptive responses on both the molecular and systemic levels (Baillieul et al., 2017; Rani et al., 2025; Burtscher et al., 2023a). Although oxygen availability is not considered to be reduced sufficiently up to about 2,500 m to induce meaningful physiological hypoxia responses in an awake state and at rest, in certain conditions, e.g., during exercise or sleep, the hypoxia occurring at moderate altitude may be strong enough (Rojas-Córdova et al., 2023). Support for meaningful physiological effects of hypoxia already at moderate altitude comes from the observation that in humans the hemoglobin concentration – which is regulated by precise oxygen sensing mechanisms – continuously and in a linear fashion increases in people living at altitudes from 200 m to 2000 m (Staub et al., 2020).

**FIGURE 1:**
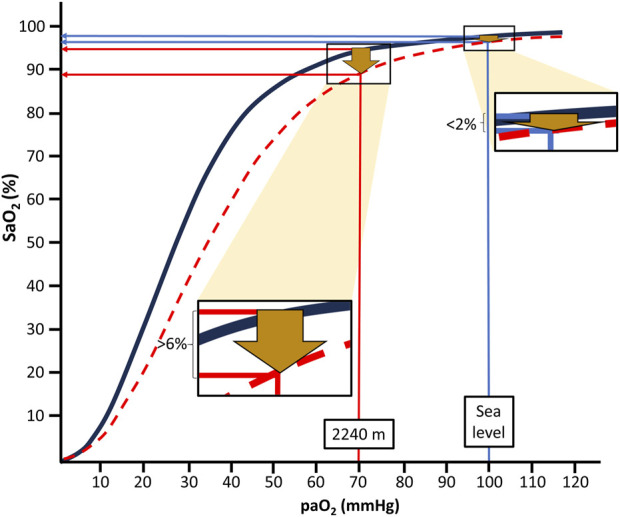
Arterial oxygen saturation (SaO_2_) values at the different levels of arterial oxygen partial pressure (PaO_2_) at sea level and moderate altitude, i.e., 2,240 m; at rest (black line) and during intense exercise (red dotted line) based on reported blood gas values and equations suggested by S.everinghaus.

The oxygen-hemoglobin dissociation curve (ODC, Figure 1) illustrates that there is an apparently negligible decline of the arterial oxygen saturation (SaO_2_) when moving from sea level to moderate altitudes, i.e., 1,000–2,500 m, in the provided example in Figure 1 of <2%. This is the case despite an expected large decrease of the arterial partial pressure of oxygen (PaO_2_) from about 100 mmHg to 70 mmHg at 2,240 m (Cid-Juárez et al., 2023). From this point (the knee of the ODC), a steep decline of SaO_2_ follows with each further drop in PaO_2_.

Depending on factors like increased temperature and decreased pH of blood, the ODC is shifted to the right during physical exercise. This results in a much more pronounced (>6%) drop in SaO_2_ already at moderate altitude (i.e., 2,240 m in the example of Figure 1) compared to sea level conditions. Thus, during exercise, SaO_2_ drops from about 95% at rest to below 90%, usually considered to indicate hypoxemia. Similarly, PaO_2_ also declines during mild hypoventilation (e.g., during normal sleep or sleep-disordered breathing), resulting in considerably more pronounced decreases of SaO_2_ at moderate altitudes than at sea level (Rojas-Córdova et al., 2023). This behavior of the ODC may be highly relevant for the more than 860 million people residing at moderate altitudes between 1,000 and 2,500 m worldwide (Tremblay and Ainslie, 2021) and also for the many millions of people annually visiting moderate altitudes for sightseeing, hiking and skiing (Burtscher et al., 2023b). Consequently, people living at or sojourning to moderate altitudes repeatedly experience physiologically relevant drops in oxygen availability, likely inducing a phenomenon similar to “hypoxia conditioning”.

## 3 Potential mechanisms of hypoxia conditioning

The concept of hypoxia conditioning has been first applied in Soviet pilots to accelerate acclimatization to high altitude (Serebrovskaya, 2002). Continuous or repeated exposures to hypoxia at an adequate “dose” (intensity and duration of the hypoxia exposure) initiate adaptive responses. Methods aiming for these benefits are termed hypoxia conditioning (Burtscher et al., 2023a; Verges et al., 2015). The dose must be strong enough (i.e., SaO_2_ below 90%) to trigger these responses but low enough for the organism to cope with the hypoxic stress without suffering injury. If these preconditions are met, e.g., during exercise or sleep at moderate altitude, hypoxia conditioning and its health-promoting effects may occur. This is indicated, for example, by the demonstrated risk reduction of cardiac arrest during exercise after sleeping one night at moderate altitude (Lo et al., 2013). The induced adaptive responses may in turn cause protection from various stressors which are linked, e.g., to acute cardiovascular events in the short-term and to the development of chronic diseases and ageing in the long-term (Burtscher et al., 2023a). Mechanistically, hypoxia conditioning is thought to be mediated by a multitude of molecular and systemic responses that can lead to beneficial adaptations, some of which are discussed below.

The discovery and characterization of molecular responses to hypoxia, notably involving the hypoxia-inducible factors (HIFs) (Kaelin Jr and Ratcliffe, 2008; Semenza, 2012), has led to a significantly improved understanding of the oxygen sensing mechanisms and hypoxia responses. In response to cellular hypoxia, these key transcription factors orchestrate the expression of multiple genes that initiate adaptive molecular processes aimed at improving oxygen supply, reducing the dependence on oxygen and increasing cellular resilience. These adaptations can have profound clinical importance. For example, intermittent hypobaric hypoxia applications were found to improve myocardial perfusion in coronary patients (del Pilar Valle et al., 2006). Even intermittent hypoxia in obstructive sleep apnea were shown to have possible cardio-protective effects, i.e., development of new coronary collateral vessels (Liu et al., 2022), although in obstructive sleep apnea the hypoxic dose usually by far exceeds the upper threshold for therapeutic hypoxia conditioning effects (Navarrete-Opazo and Mitchell, 2014). Many cellular components other than HIFs contribute to the molecular responses to hypoxia. Among them, the transcription factor nuclear factor erythroid 2-related factor 2 (Nrf2), a core regulator of cellular responses to oxidative stress, importantly contributes to health-promoting effects of hypoxia conditioning, including cardiovascular and central nervous system benefits (Burtscher et al., 2023a). During the transition from hypoxia to normoxia (hypoxia-reoxygenation), reactive oxygen species (ROS) are generated, activating Nrf2 (Burtscher et al., 2022). In turn, Nrf2 promotes the upregulation of antioxidant pathways and protects from related cell and tissue damage (Sprick et al., 2019). Thus, hypoxia-reoxygenation events, as occurring during sleep or exercise at moderate altitude, may represent major inducers of antioxidant defense mechanisms, promoting cellular, tissue and organism resilience (Sprick et al., 2019). Oxidative stress, like the associated mitochondrial dysfunction and inflammation, are crucial factors in the promotion of (age-related, including metabolic, cardiovascular and neurological) diseases and aging itself, which may be partially counteracted by hypoxia conditioning (Burtscher et al., 2023a).

Systemic responses and adaptations to hypoxia include respiratory facilitation, modulation of coronary and cerebral blood flow, attenuation of stress-related sympatho-excitation, regulation of appetite and favorable metabolic adaptations, and improvement of exercise tolerance (Glazachev et al., 2021). All these adaptations may contribute to the lower cardiovascular mortality when living at moderate altitude (Burtscher et al., 2021; Ezzati et al., 2012; Faeh et al., 2009). Some of them such as lower sympatho-adrenergic-activity have been suggested as potential explanations for the observed risk reduction to suffer from sudden cardiac death during physical activity, i.e., skiing, after short moderate altitude exposures (Lo et al., 2013).

We propose that some conditions, like exercising or sleeping, aggravate the drop in SaO_2_ at moderate altitude to levels sufficient for the induction of clear physiological responses even to the mild ambient hypoxia. As illustrated in the presented example (Figure 1), a moderate right-shift of the ODC (e.g., through exercise) can push the SaO_2_ down to levels (about 92%–90%) that have been linked to robust systemic and molecular (for example, upregulation of HIF-regulated erythropoietin) hypoxia responses (Ge et al., 2002).

## 4 Conclusion

Life at moderate altitude and the related intermittently occurring drops in SaO_2_ may represent natural hypoxia conditioning scenarios that increase the resilience of the human organism. This in turn may contribute to the risk reduction of acute cardiovascular adverse events and in particular that of dying from chronic diseases such as cardiovascular diseases and certain cancers.
